# A Three-Dimensional Bioprinted Copolymer Scaffold with Biocompatibility and Structural Integrity for Potential Tissue Regeneration Applications

**DOI:** 10.3390/polym14163415

**Published:** 2022-08-21

**Authors:** Bou-Yue Peng, Keng-Liang Ou, Chung-Ming Liu, Shu-Fen Chu, Bai-Hung Huang, Yung-Chieh Cho, Takashi Saito, Chi-Hsun Tsai, Kuo-Sheng Hung, Wen-Chien Lan

**Affiliations:** 1 School of Dentistry, College of Oral Medicine, Taipei Medical University, Taipei 110, Taiwan; 2 Division of Oral and Maxillofacial Surgery, Department of Dentistry, Taipei Medical University Hospital, Taipei 110, Taiwan; 3 Biomedical Technology R & D Center, China Medical University, Taichung 404, Taiwan; 4 Department of Dentistry, Taipei Medical University-Shuang Ho Hospital, New Taipei City 235, Taiwan; 5 Division of Clinical Cariology and Endodontology, Department of Oral Rehabilitation, School of Dentistry, Health Sciences University of Hokkaido, Hokkaido 061-0293, Japan; 6 3D Global Biotech Inc. (Spin-off Company from Taipei Medical University), New Taipei City 221, Taiwan; 7 Taiwan Society of Blood Biomaterials, New Taipei City 221, Taiwan; 8 Graduate Institute of Dental Science, College of Dentistry, China Medical University, Taichung 404, Taiwan; 9 College of Nursing and Health Management, Shanghai University of Medicine and Health Sciences, Shanghai 201318, China; 10 Graduate Institute of Injury Prevention and Control, College of Public Health, Taipei Medical University, Taipei 110, Taiwan; 11 Department of Neurosurgery, Taipei Medical University-Wan Fang Hospital, Taipei 116, Taiwan; 12 Department of Oral Hygiene Care, Ching Kuo Institute of Management and Health, Keelung 203, Taiwan

**Keywords:** hydrogel, printability, bioink, cell viability, rheological property

## Abstract

The present study was to investigate the rheological property, printability, and cell viability of alginate–gelatin composed hydrogels as a potential cell-laden bioink for three-dimensional (3D) bioprinting applications. The 2 g of sodium alginate dissolved in 50 mL of phosphate buffered saline solution was mixed with different concentrations (1% (0.5 g), 2% (1 g), 3% (1.5 g), and 4% (2 g)) of gelatin, denoted as GBH-1, GBH-2, GBH-3, and GBH-4, respectively. The properties of the investigated hydrogels were characterized by contact angle goniometer, rheometer, and bioprinter. In addition, the hydrogel with a proper concentration was adopted as a cell-laden bioink to conduct cell viability testing (before and after bioprinting) using Live/Dead assay and immunofluorescence staining with a human corneal fibroblast cell line. The analytical results indicated that the GBH-2 hydrogel exhibited the lowest loss rate of contact angle (28%) and similar rheological performance as compared with other investigated hydrogels and the control group. Printability results also showed that the average wire diameter of the GBH-2 bioink (0.84 ± 0.02 mm (*** *p* < 0.001)) post-printing was similar to that of the control group (0.79 ± 0.05 mm). Moreover, a cell scaffold could be fabricated from the GBH-2 bioink and retained its shape integrity for 24 h post-printing. For bioprinting evaluation, it demonstrated that the GBH-2 bioink possessed well viability (>70%) of the human corneal fibroblast cell after seven days of printing under an ideal printing parameter combination (0.4 mm of inner diameter needle, 0.8 bar of printing pressure, and 25 °C of printing temperature). Therefore, the present study suggests that the GBH-2 hydrogel could be developed as a potential cell-laden bioink to print a cell scaffold with biocompatibility and structural integrity for soft tissues such as skin, cornea, nerve, and blood vessel regeneration applications.

## 1. Introduction

The increasing demand for tissues or organs that meet the criteria to replace damaged or lost tissue or organ functions makes tissue engineering an encouraging technique to shape human organs and tissues [1,2,3]. Although the formation of the two-dimensional scaffold was successful in vitro through tissue engineering, it was inadequate to resemble the original tissue in a complex manner, nor did the perspective of three-dimensional (3D) polymer scaffolding [1,4]. Therefore, the 3D bioprinting approach has been developed to overcome various shortcomings of tissue engineering, especially in the formation of a stable scaffold with biocompatibility for cell survival, which allows the fabrication of multi-cellular tissues needed in copious tissue engineering applications [1,5,6,7,8,9,10,11]. Forming organs or tissues that are following the complex microarchitecture of native tissue through a bioprinting approach face various challenges in overcoming the low level of biocompatibility to cells, which leads to a loss or damage to cell function, as well as blockages during the printing process [1,2,12,13,14]. The very complex structure possessed by native tissues is predominant for carrying out specific functions, making bioinks with a single biomaterial unable to meet the criteria both mechanically and functionally to produce tissue biomimetics [7,15].

Natural polysaccharides such as cellulose, chitosan, chitin, alginate, gelatin, hyaluronic acid, and polyethylene glycol derivatives can be combined with other biomaterials to form potential composites showing improved properties [16,17,18,19,20,21,22,23]. These potential composites have also been applied in 3D cell microenvironments through the hydrogel system [11,17,24,25]. Alginate and gelatin are among the most commonly used hydrogel bioinks for extrusion-based printing to recreate solid tissue-like physiological models [25,26,27,28,29]. Sodium alginate hydrogel has been used in 3D bioprinting for several years because of its good biocompatibility and low cost, as well as because it can be ionically cross-linked by divalent cations to provide matrix integrity at a physiological temperature [17,30]. The shear-thinning properties and structure resembling an extracellular matrix favor the use of alginates in the manufacture of bioinks [2]. The inclusion of calcium ions directs cross-linking of the carboxylate groups of sodium alginate to achieve excellent gelation [1,31]. During the gelation process, calcium will diffuse into alginic acid, which will cause a decrease in pH and have an impact on cell damage [1]. Pure alginate hydrogels in the manufacture of bioinks also have low solution viscosities with the resulting filaments being easily degraded and disintegrated, making the solid structure of 3D bioprinting difficult to obtain [2]. Moreover, alginate is known to have no motif to facilitate cell attachment [1,31,32]. Gelatin is known to improve mechanical properties, good printability, and increase cell attachment [9,18,31,33]. Because gelatin is a derivative of collagen, the use of gelatin is more economical than pure collagen and does not have collagen antigens to reduce the potential of immune rejection [4,31]; while gelatin is also not used alone for bioprinting because it is highly temperature dependent and the reversible sol-gel transition makes it problematic to modify the printing temperature and viscosity [4]. Therefore, it is believed that multicomponent bioinks in the case of alginate–gelatin mixtures can not only enhance printability but can also improve mechanical properties and cell viability to obtain the desired bioprinting results [2,31].

However, there is a lack of reports to show the structural integrity of the multi-layer printed alginate–gelatin scaffolds without any reinforced biomaterials addition can be maintained for a long period post-printing. It is also a vital challenge in soft tissue engineering to provide a stable and complete printed gel-like scaffold for cell growth to form the desired soft tissues such as skin, cornea, nerve, and blood vessels. To solve this issue, the present study investigated the printability of the alginate–gelatin hydrogel as bioink. It is well known that the concentration of gelatin solutions can result in significantly different flowability and printability. Thus, we determined the stable printing quality and developed a preliminary ideal index to print a complex scaffold without overhang using different concentrations of gelatin via a self-assembled bioprinter. Based on the investigated parameters, the present study could provide a potential solution to print the alginate–gelatin-composed complex scaffold that mimics natural tissues/organs for soft tissue engineering and organ regenerative applications.

## 2. Materials and Methods

### 2.1. Hydrogels Preparation

The 2 g of sodium alginate (187 kDa, Sigma W201502, Taipei, Taiwan) was dissolved with 50 mL of phosphate buffered saline (PBS, Sigma, Taipei, Taiwan) solution in a 250 mL beaker, and then stirred with a magnetic stirring bar at 60 °C for 2 h. The dissolved sodium alginate was following placed into a 50 mL of centrifuge tube. Subsequently, the gelatin (50–100 kDa, Sigma G2500, Taipei, Taiwan) with different concentrations (1% (0.5 g), 2% (1 g), 3% (1.5 g), and 4% (2 g)) was added in the dissolved sodium alginate, respectively. Then, the mixture solution was centrifuged to remove tiny bubbles at 1500 rpm for 10 min. For easy classification, the resulting hydrogel is denoted according to the concentration of gelatin added, namely, GBH-1, GBH-2, GBH-3, and GBH-4. Calcium chloride (Sigma, C7902, Taipei, Taiwan) was dissolved in PBS solution until it reached 50 mM as a cross-linking agent. Before being used for printing, the hydrogels were kept at a temperature of 25 °C for 1 h. In this study, the commercial bioink product (Cellink, Gothenburg, Sweden) was used as a control group for comparison.

### 2.2. Contact Angle Analysis

The hydrophilicity of the investigated hydrogels was measured using the contact angle measurement (*n* = 5). The investigated hydrogels were used as the test substance (not water or other solvents) in this experiment. Approximately 0.05 mL hydrogel was dropped onto the surface of slide glass at a constant distance of 10 mm, and the contact angle was measured using a GBX Digidrop goniometer (GBX Scientific LTD., Romans-sur-Isère, France). The temperature and humidity test conditions were maintained at 25 °C and 60% relative humidity, respectively.

### 2.3. Rheological Property Evaluation

Rheological analysis of each hydrogel was performed using an MCR-302 rheometer (Anton Paar Instrument, Graz, Austria) supplied with a 20 mm parallel plate geometry and a measurement gap of 0.2 mm. Before testing, 2 mL of hydrogel was placed on the lower plate of the rheometer and kept at 25 °C for 5 min. Excess hydrogel around the trim gap was removed with a spatula. Thereafter, flow rate sweeps were conducted with a shear rate in the range of 0.002 to 500 s^−1^ at 25 °C. An average of triplicates per hydrogel was adopted in the rheological measurement.

### 2.4. Filament Fusion Testing

**Figure 1:**
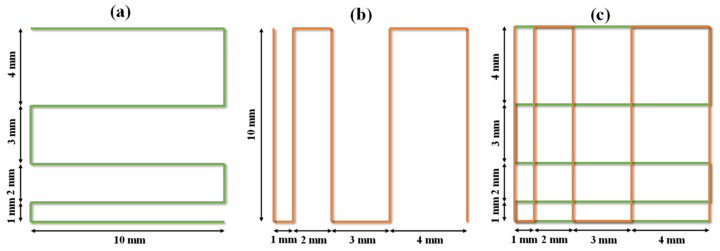
Filament fusion testing pattern printed along: (**a**) 1st layer, (**b**) 2nd layer, and (**c**) final pattern.

A self-assembled 3D bioprinter with an extruded syringe dispenser system was utilized to evaluate the printability of the investigated hydrogels. Filaments were printed along 0° (1st layer) and 90° (2nd layer) as a pattern with a filament distance of 1.0 mm, 1.0 mm increments for each subsequent line, and finishing filament at the distance of 4.0 mm (Figure 1). Five mm s^−1^ of print speed and a 22-gauge needle (inner diameter of 0.4 mm) were used in this test. In addition, the printing pressure of GBH-1, GBH-2, GBH-3, GBH-4, and control were 0.4 bar, 0.8 bar, 1.5 bar, 2.5 bar, and 0.5 bar, respectively. The printing temperature was 25 °C. To prevent undesirable material spreading, the fabricated scaffold was captured immediately after fabrication with a high-resolution camera. Wire diameter measurement and the plotted values represent six repetitions of measurements.

### 2.5. Filament Collapse Testing

**Figure 2:**
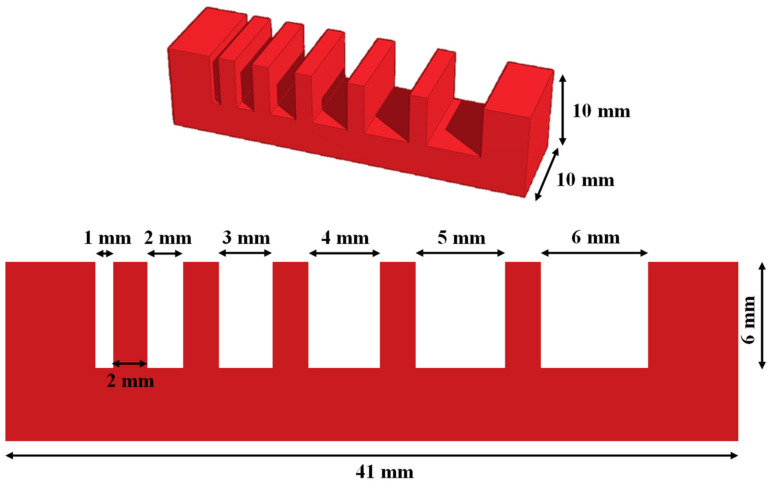
Model of the platform for filament collapse testing.

Filament collapse testing used five pillars in the middle (2 × 10 × 6 mm^3^) and two pillars in the end (5 × 10 × 6 mm^3^), with known gap distances of 1, 2, 3, 4, 5, and 6 mm between each other (Figure 2). The platform was fabricated using a “Form 2” 3D printer made by Formlabs Inc. (Somerville, MA, USA). This test used 5 mm s^−1^ of print speed and a 22-gauge needle. The printing pressure of GBH-1, GBH-2, GBH-3, GBH-4, and control were 0.4 bar, 0.8 bar, 1.5 bar, 2.5 bar, and 0.5 bar, respectively. The printing temperature was 25 °C. To keep away from unfavorable material deflection, the printed filament was captured immediately after suspension using a high-resolution camera.

### 2.6. Cell Viability Assay

**Figure 3:**
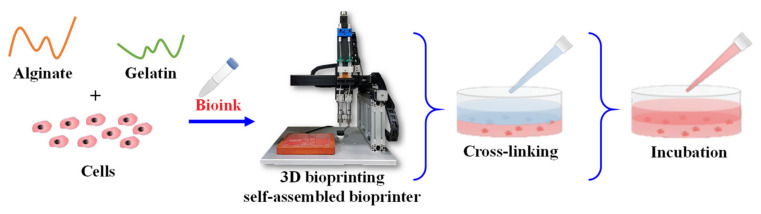
A schematic diagram showing the experimental setup used for evaluation of the post-printing cell viability of the optimal bioink.

In this study, the human corneal fibroblast cell line (no. 6510, Blossom Biotechnologies Inc., Taipei, Taiwan) was adopted to evaluate the cell viability of the optimal bioink before (without printing treatment) and after printing (with printing treatment). For the cell viability before printing, the fibroblast suspension (4 × 10^5^ cells) and 1 mL bioink were loaded into a 35 mm dish, and 1 mL of 50 mM CaCl_2_ solution as a cross-linking agent was added and soaked for 5 min (*n* = 5). When the cross-linking was complete, CaCl_2_ solution was removed and washed 3 times with PBS solution. Hereafter, 1 mL of culture medium (DMEM/F12) was added and incubated at 37 °C with 5% CO_2_ for 1 day and 7 days, respectively. For the post-printing cell viability evaluation (Figure 3), The 1 mL bioink with a concentration of 4 × 10^5^ cells/mL was pipetted in a 10 mL syringe and immediately moved to an electronic dry oven with a temperature of 25 °C for 10 min to form the cell-laden bioink. Afterward, a 200 μL cell-laden bioink was printed into a 35 mm dish (*n* = 5) according to the ideal printing combination as mentioned in Section 2.4. Subsequently, a 1 mL of 50 mM CaCl_2_ solution as a cross-linking agent was added, soaked for 5 min. After the cross-linking was complete, CaCl_2_ solution was removed, washed by PBS solution three times. Hereafter, 1 mL of culture medium was added and incubated at 37 °C and 5% CO_2_ for 1 day and 7 days, respectively. At the end of each time point, the LIVE/DEAD^®^ Viability/Cytotoxicity Kit fluorescent dyes (488/570; Thermo Fisher Scientific, Paisley, UK) was added and incubated at 37 °C with 5% CO_2_ for 1 h. Finally, the cells with printing treatment on day 1 and day 7 were observed through an inverted fluorescence microscope (Olympus IX71, Tokyo, Japan) under different magnifications. For the quantification of cells at each time point, images were acquired using the VisiView software (Visitron Systems GmbH, Puchheim, Germany). Then, red fluorescent cells (dead cells) were counted in representative images acquired from 3 to 4 randomly. The numbers of dead cells were assessed using the Fiji-ImageJ image processing software, which permits practicable cell counting. The cell viability is expressed as shown in Equation (1):

(1) $$\mathrm{Cell}\;\mathrm{viability}\;(\%)=(1-\frac{\mathrm{Dead}\;\mathrm{cell}}{\mathrm{Total}\;\mathrm{cell}})\times 100\%$$

### 2.7. Statistical Analysis

The experimental data were analyzed via SPSS statistic software (Version 19.0., SPSS Inc., Chicago, IL, USA). The difference between multiple groups were determined by one-way analysis of variance followed by Tukey’s HSD post hoc test. A *p* value less than 0.05 was considered statistically significant.

## 3. Results

### 3.1. Wettability Property

**Figure 4:**
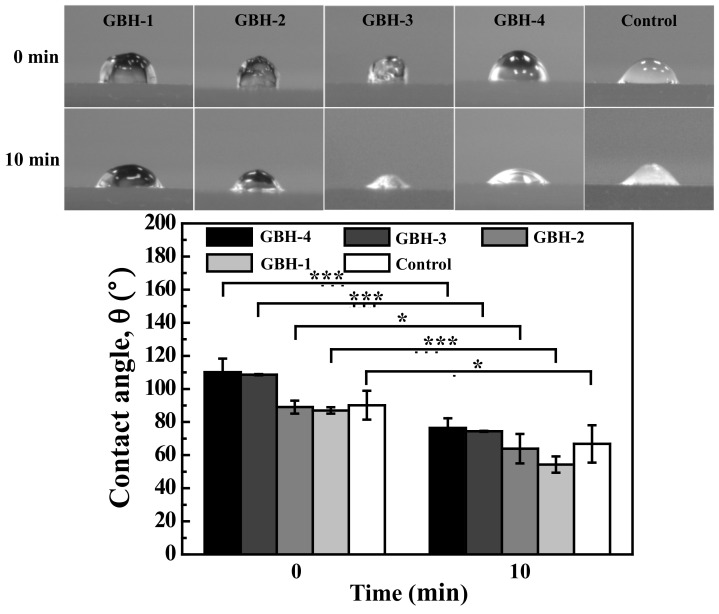
Contact angle results of the investigated hydrogels (* *p* < 0.05 and *** *p* < 0.001).

The contact angle will affect the printability test. If the contact angle is reduced a lot, it will collapse quickly during 3D printing. It was found that the loss rate of the GBH-4, GBH-3, and GBH-1 hydrogels was around 30%, which was higher than that of the control hydrogel with 26% after contact of 10 min (Figure 4). However, the GBH-2 hydrogel possessed the lowest loss rate of 28%, which was similar to the control hydrogel. The quantitative analysis revealed the loss rate of the GBH-2 hydrogel increased overtime which could cause the degree of collapse to be low in 3D printing.

### 3.2. Rheological Variation

**Figure 5:**
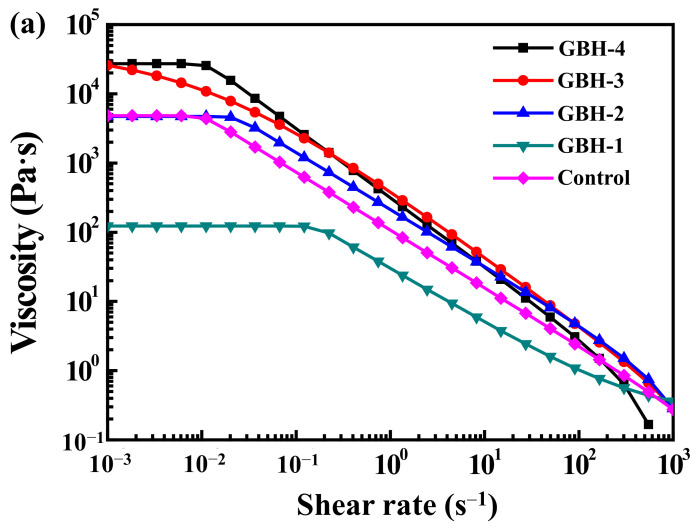

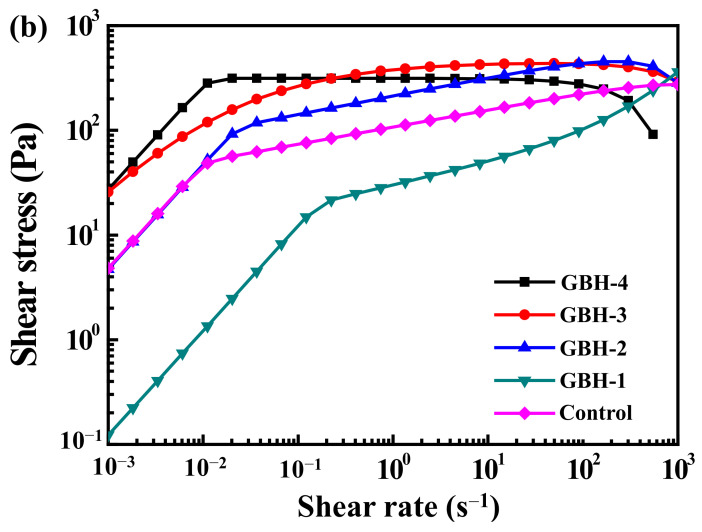
The rheological properties of the investigated hydrogels: (**a**) viscosity and (**b**) shear stress.

The rheological properties of the investigated hydrogels as displayed in Figure 5. At a low shear rate, the viscosity of all hydrogels decreased with increasing shear rate, suggesting the shear thinning behavior (Figure 5a) leads to bioink fluent crossing the nozzle with minimal plugging. The relationship curve between shear stress–shear rate is inversely proportional to the viscosity–shear rate curve (Figure 5b) because higher viscosity needs higher pressure to release bioink from the nozzle. Moreover, the viscoelasticity of the GBH-2 hydrogel was similar to that of the control hydrogel.

### 3.3. Printability Characterization

**Figure 6:**
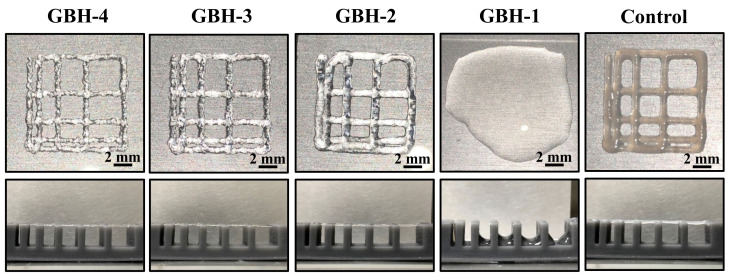
Filament fusion (**upper** line) and collapse testing (**bottom** line) results of the investigated hydrogels.

**Figure 7:**
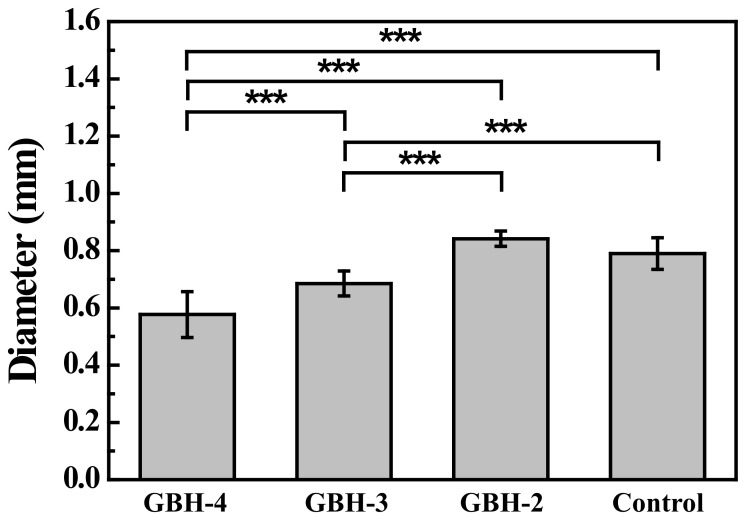
Measuring wire diameter by filament fusion testing (*** *p* < 0.001).

**Figure 8:**
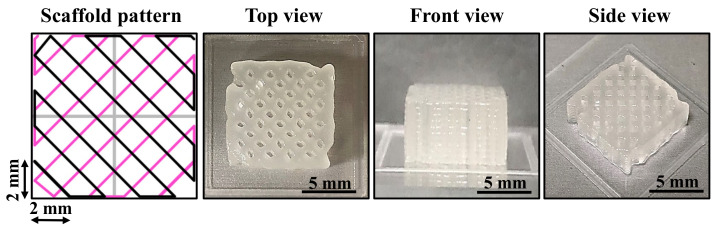
The shape integrity of a cell scaffold (10 mm × 10 mm × 10 mm) from the GBH-2 bioink was maintained for 24 h post-printing. The grid pattern spacing was 2 mm and the print layer was 10 layers.

The filament fusion and collapse testing results of the investigated bioinks after printing as shown in Figure 6. It is clearly seen that the GBH-2 bioink exhibited a well-aligned pattern after printing as compared with the control bioink. A similar result can also be found in filament collapse testing. Figure 7 presents the measurement of wire diameter testing results. Obviously, the average wire diameter of the GBH-2 bioink (0.84 ± 0.02 mm (*** *p* < 0.001)) was similar to that of the control bioink (0.79 ± 0.05 mm). Furthermore, it was found that the cell scaffold printed by GBH-2 bioink was stable and did not collapse after 24 h of printing (Figure 8). These printing results revealed that the GBH-2 bioink has stable printability potential.

### 3.4. Live/Dead Staining Assessment

**Figure 9:**
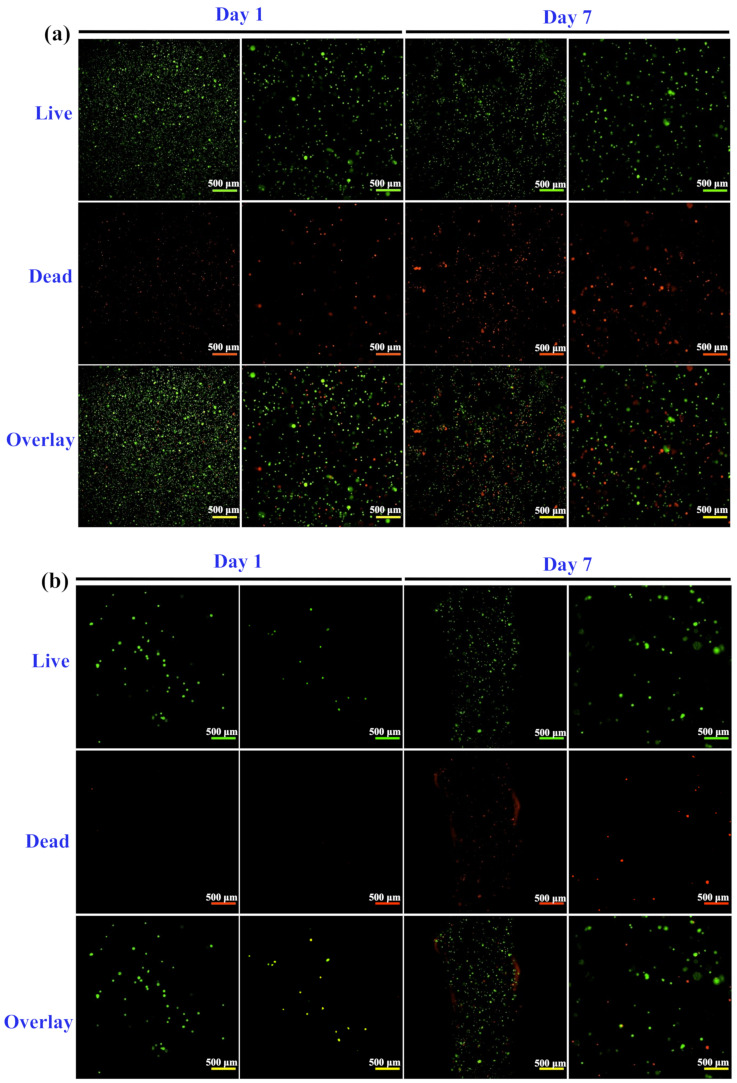

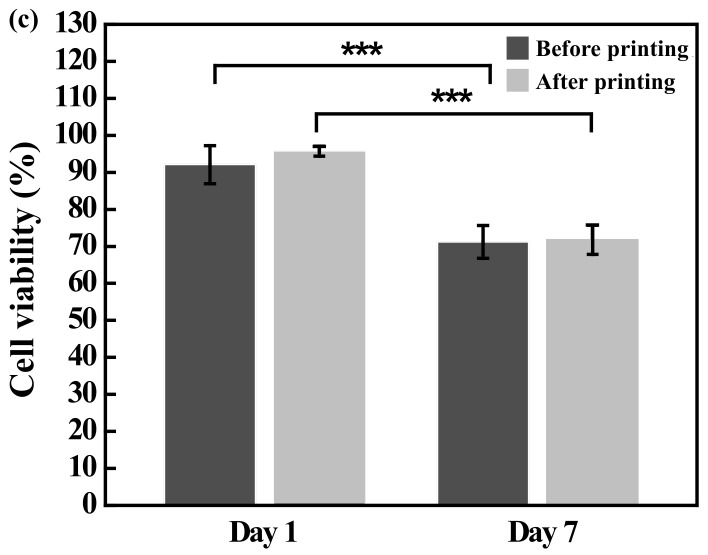
Live/Dead analysis results of cell viability of the optimal bioink GBH-2: (**a**) before printing (without printing treatment), (**b**) after printing (with printing treatment), and (**c**) quantitative measurement analysis (*** *p* < 0.001).

Figure 9 displays the Live/Dead analysis of the optimal bioink GBH-2. Cell viability of the GBH-2 bioink both before (Figure 9a) and after printing (Figure 9b) showed the highest amounts of live cells on day 1 than on day 7. Additionally, quantitative analysis of cell viability proved that there was statistically different cell viability before and after printing on day 1 and day 7 (*** *p* < 0.001) (Figure 9c). Although, the cell viability was decreased on day 7, the cell viability was still higher than 70%. This result demonstrated that after bioprinting with cell-laden GBH-2 bioink, it presented a slight effect on the cell viability of human corneal fibroblast cells only.

## 4. Discussion

The printability of hydrogels is a key aspect in providing a suitable environment to create complex tissue/organ structures for organ functionalization using 3D bioprinting technology [2,8,15,18]. In the present study, we proposed a preliminary ideal index of the alginate–gelatin composed hydrogel as a potential cell-laden bioink to generate 3D architectures through a self-fabricated 3D bioprinting system. It was found that the alginate mixed with 2% of gelatin (GBH-2) bioink was able to print a bridge with straight filament between 2 pillars in the collapse test, and in the fusion test, it produced an excellent geometry with minimum spreading similar to the control bioink. The properties of the GBH-2 bioink are most likely because the material is not too viscous in under-gelation conditions [34]. The combination of bioink alginate and gelatin seems to have great potential in producing a hybrid scaffold with increased printability and mechanical and biological properties [2,31,32]. This feature can be attributed to the change in the wettability (contact angle) and viscoelasticity of the investigated GBH-2 bioink. The contact angle can affect the accuracy of bioink printing [3,35]. The low surface energy of the printing substrate obtained from the hydrophobic material allows the bioink not to spread [3,6,18,31,36]. Meanwhile, hydrophobicity is required to anchor the printed construction to the receiving surface, minimizing unwanted movement and possible deformation during the printing process with bioink layer by layer [31]. Therefore, to get good printability results, the contact angle reduction should be gradual during the printing process starting from the first layer of printing [31]. In this study, it could be identified in the hydrophobic value limit, but over time, the contact angle also shows a little hydrophilicity; this result is in line with the study on making on-chip organs using 3D bioprinting technology, which demonstrates better print results, but is able to support cell function [37,38,39].

Moreover, the viscoelasticity value is related to the extrusion of the bioink, which affects its spread [31,40]. Bioink with high viscosity may prevent spread, yet a viscosity that is too high requires high pressure to encourage bioink extrusion and will have an impact on disrupting cell viability [12,22,31,36,41,42,43]. Therefore, the viscosity control of the bioink is very important. The results of the current study show that among the investigated bioink concentrations, the GBH-2 bioink is analogous to competitors with a viscosity that is neither too high nor too low. These properties proved appropriate in the context of better printability; while the preliminary experiment to study the viability of human corneal fibroblasts combined with GBH-2 bioink within the ideal printing process indicated that more cell death was present 7 days post-printing. The decrease in post-printing cell viability is the commonly encountered issue in 3D bioprinting applications [44]. This might be caused by the pressure used to expel the bioink in micro-extrusion bioprinting [31,45]. Printing at higher extrusion pressures will increase the number of dead cells since a higher pressure will elevate the shear stress in the nozzle, which eventually damages the cell membrane and leads to lower cell viability after extrusion [46]. Bishop et al. [47] have also reported an inverse relationship between extrusion pressure and cellular viability, especially in high extrusion pressures. However, in the analysis of cell viability of GBH-2 bioink, cells still survive up to 7 days post-printing and possessed a survival rate of 70%. This finding revealed that the GBH-2 hydrogel might be a favorable bioink to stimulate cell growth and proliferation when formulated for cell-encapsulation in 3D bioprinting. Referring to the results of the analysis of contact angle, viscoelasticity, collapse, and fusion, and cell viability assay, the present study suggested that the GBH-2 bioink is a promising candidate for producing good printability, shape integrity, and an environment that is not harmful to cell survival. However, more experiments must be performed to validate the present findings.

## 5. Conclusions

The GBH-2 hydrogel showed the lowest contact angle loss rate of 28% during the printing process in comparison to other investigated hydrogels. Rheological properties of all hydrogels exhibited shear thinning behavior. However, the GBH-2 hydrogel possessed similar viscosity, low shear stress, and average wire diameter post-printing to the control group. The combination of alginate and gelatin in the GBH-2 revealed great potential in creating a cell scaffold to retain its shape integrity for 24 h post-printing. In addition, the GBH-2 bioink also demonstrated excellent printability with maintaining a survival rate (>70%) of the human corneal fibroblast cell before and after printing. Thus, the present findings could provide a new strategy to generate a complex configuration of a functional cell scaffold for soft tissue engineering applications.

## Data Availability

Data are contained within the article.
